# Proto-oncogene HER-2 in normal, dysplastic and tumorous feline mammary glands: an immunohistochemical and chromogenic in situ hybridization study

**DOI:** 10.1186/1471-2407-7-179

**Published:** 2007-09-20

**Authors:** Javier Ordás, Yolanda Millán, Rafaela Dios, Carlos Reymundo, Juana Martín de las Mulas

**Affiliations:** 1 Departamento de Anatomía Patológica Comparada, Facultad de Veterinaria, Universidad de Córdoba, Campus Universitario de Rabanales, Carretera de Madrid- Cádiz Km 396 14014 Córdoba, Spain; 2 Departamento de Estadística, Econometría, Investigación Operativa y Organización de Empresas, E.T.S. Ingenieros Agrónomos y de Montes, Universidad de Córdoba, Campus Universitario de Rabanales, Carretera de Madrid-Cádiz Km. 396 14014 Córdoba, Spain; 3 Departamento de Anatomía Patológica, Facultad de Medicina, Universidad de Córdoba, Avda. Menéndez Pidal s/n, 14001 Córdoba, Spain

## Abstract

**Background:**

Feline mammary carcinoma has been proposed as a natural model of highly aggressive, hormone-independent human breast cancer. To further explore the utility of the model by adding new similarities between the two diseases, we have analyzed the oncogene HER-2 status at both the protein and the gene levels.

**Methods:**

Formalin-fixed, paraffin-embedded tissue samples from 30 invasive carcinomas, 7 benign lesions and two normal mammary glands were analyzed. Tumour features with prognostic value were recorded. The expression of protein HER-2 was analyzed by immunohistochemistry and the number of gene copies by means of DNA chromogenic *in situ *hybridization.

**Results:**

Immunohistochemical HER-2 protein overexpression was found in 40% of feline mammary carcinomas, a percentage higher to that observed in human breast carcinoma. As in women, feline tumours with HER-2 protein overexpression had pathological features of high malignancy. However, amplification of HER-2 was detected in 16% of carcinomas with protein overexpression, a percentage much lower than that observed in their human counterpart.

**Conclusion:**

Feline mammary carcinoma would be a suitable natural model of that subset of human breast carcinomas with HER-2 protein overexpression without gene amplification.

## Background

Human epidermal growth factor receptor type 2 (HER-2), alias c-erbB-2 and neu, is a protooncogene that encodes a transmembrane glycoprotein similar to the human epidermal growth factor receptor known as the HER-2 protein. HER-2 has been described in different tumors and animals. In rats and mice, transforming activity of the neu oncogene is associated with somatic mutations [1]. In humans, the abnormal (high) expression of HER-2 protein (so-called overexpression) correlates with more aggressive clinicopathologic features, drug resistance or sensitivity to specific chemotherapy and specific hormonal therapy regimens in breast cancer [2]. HER-2 protein overexpression is found in 15–30% of human breast carcinomas and comparative fluorescent *in situ *hybridization studies have shown that gene amplification is present in some 85–90% of the cases [2,3]. Chromogenic *in situ *hybridization (CISH) has been shown to have good correlation with FISH [4,5], which is currently regarded as a gold standard method for detecting HER-2 amplification, but it is not very practical for routine histopathological laboratories.

Feline and canine mammary tumours have epidemiological, clinical, morphologic and prognostic features similar to those of human breast carcinoma, for which they are suitable natural models [6,7]. However, similarities concerning both histological picture and biological behavior are higher in feline cases because, contrary to the situation in dogs, histological evidence of malignancy is present in more than 80% of the cases and associates with an aggressive clinical course [8]. Alterations of the HER-2 proto-oncogene have been described in mammary tumors of cats [9-11] and dogs [12-16] mostly at the protein level. The aim of the present work was to investigate the alterations of proto-oncogen HER-2 in normal and tumorous feline mammary glands at both the protein and gene levels to further explore the value of feline mammary carcinomas as natural models of human breast carcinomas.

## Methods

Formalin-fixed paraffin-embedded tissue samples from 30 invasive simple epithelial carcinomas, 7 benign lesions (5 fibroepithelial hyperplasia and 2 simple adenoma) [17] and two normal mammary glands were analyzed. Data regarding tumor size and histologic grade of malignancy [18] were recorded. The immunohistochemical expression of estrogen receptor α (ERα) and progesterone receptor (PR) was analysed as described previously [19,20].

### Protein HER-2 expression

**Figure 1:**
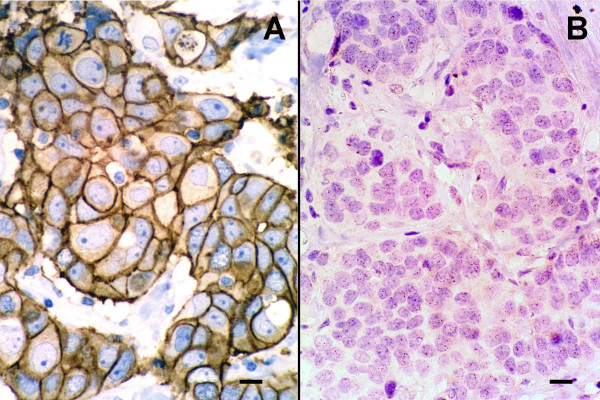
HER-2 protein expression and oncogene copies in human breast carcinoma. A) Infiltrating duct carcinoma with HER-2 protein overexpression scored (+++). HercepTest, scale bar = 5 μm. B) The same tumour shows more than 5 gene copies/nucleus in more than 50% of cancer cells indicating oncogene amplification. CISH, scale bar = 5 μm.

A commercial polyclonal antibody anti-HER-2/neu protein (Dakocymation, Glostrup, Denmark) diluted 1:1000 and the avidin-biotin-peroxidase immunohistochemical method (ABC, Vector, Burlingame, CA) were applied to deparaffined and dehydrated tissue sections after high temperature antigen retrieval as described elsewhere [14]. Samples of human breast carcinoma that had been scored as positive (+++) or negative (-/+) with the HercepTest™ (Dakocymation, Glostrup, Denmark) were used as positive and negative controls, respectively (Figure 1A). The results were scored according to the criteria specified in the HercepTest™ as follows: 0 = no staining or weak and incomplete membrane staining in less than 10% of the neoplastic cells; (+) = incomplete and faint membrane staining in more than 10% of the neoplastic cells; (++) moderate and complete membrane staining in more than 10% of the neoplastic cells; (+++) = strong and complete membrane staining in more than 10% of the neoplastic cells. According to the criteria described above, HER-2 protein overexpression is determined in cases scored as (++) and (+++).

### HER-2 oncogene status

A commercial digoxigenin (DIG)-labeled HER2 DNA probe generated by Subtraction Probe Technology (SPT™) was used following manufacturer's recommendations (Zymed Lab. Inc.). Gene copies visualized by CISH were distinguished with × 40 and/or × 100 objectives as brown dots in hematoxylin-stained tissue sections. Positive controls for the standardization of the technique included formalin-fixed paraffin-embedded tissue samples from human breast carcinomas which had been scored HercepTest™ (Dako) (+++) positive and had shown HER-2 oncogene amplification by CISH (more than 5 gene copies/nucleus or large cluster of amplification/nucleus in more than 50% of cancer cells) (Figure 1B) [4]. Equally processed tissue samples from non-altered feline mammary gland were run as negative controls in every assay. Gene detection on feline tissue samples was indicated by the presence of one to four copies in more than 80% of the nuclei.

### Statistical study

The association between HER-2 protein overexpression and tumour size as well as histological grade of malignancy and steroid hormones receptors content was assessed by the Chi-square test. P values < 0.05 were considered to reflect statistical significance.

## Results

### Protein HER-2 expression

**Figure 2:**
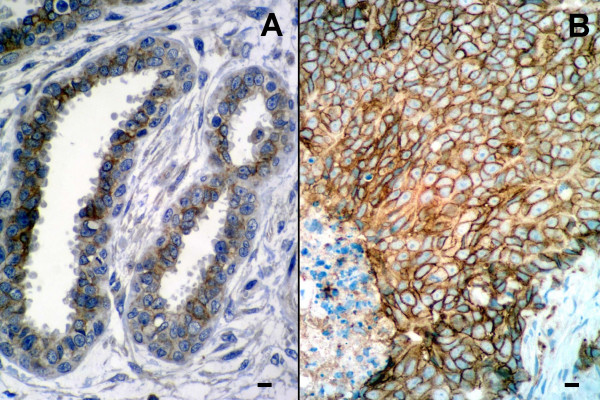
HER-2 protein expression in feline mammary gland. A) Fibroepithellial hyperplasia: some duct epithelial cells showed faint to moderate staining in part of the membrane. ABC, scale bar = 10 μm. B) Invasive mammary carcinoma: HER-2 protein overexpression scored (+++). ABC, scale bar = 20 μm.

The HER-2 polyclonal antibody raised against the human antigen crossreacted with feline tissues as a low percentage of epithelial cells of non-altered ducts and acini from normal mammary glands showed a faint, barely perceptible staining in part of the cell membrane. A similar staining pattern (+ scoring) was also observed in 2 benign lesions classified as fibroepithelial hyperplasia (Figure 2A). Protein overexpression (++ and +++ scorings) was detected in 12 out of 30 carcinomas (40%) (Figure 2B). Carcinomas with HER-2 overexpression measured more than 2 cm in their largest diameter, had the highest histologic grade of malignancy (grade III) (p = 0.011) and lacked estrogen and progesterone receptors (p = 0.046).

### HER-2 oncogene status

**Figure 3:**
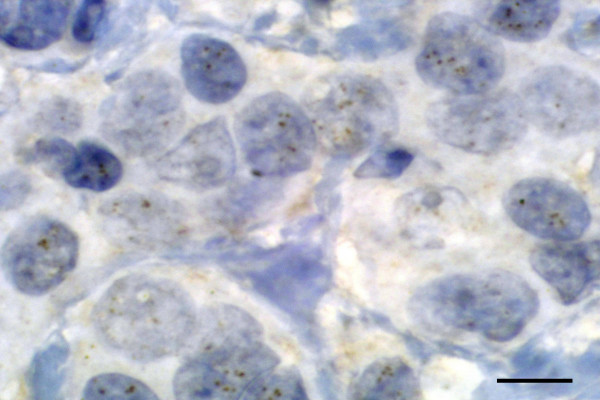
HER-2 oncogene copies in feline mammary carcinoma. Between 5 and 10 brown dots per nucleus are seen in the majority of cancer cell nuclei. CIHS, scale bar = 4 μm.

One to four brown dots per nucleus were visualized under bright-field microscope in hematoxylin-counterstained sections in more than 80% of the nuclei in tissue samples from normal mammary gland, benign proliferative lesions, all 18 carcinomas without protein overexpression as well as 10 carcinomas with HER-2 protein overexpression. The remaining 2 carcinomas with HER-2 protein overexpression had more than five and less than ten dots per nucleus (Figure 3). Accordingly, 16.6% of carcinomas with HER-2 protein overexpression had gene amplification.

## Discussion

Feline mammary carcinomas are, like human breast cancers, spontaneous, locally infiltrative and metastasizing tumors. Therefore, this tumor disease in the cat can serve as pathogenic and experimental-therapeutic model for the human counterpart [6]. The present study adds new similarities to widen the utility of the model as it shows that feline mammary carcinomas overexpress HER-2 protein as human breast cancers do [2]. In addition, feline carcinomas with HER-2 overexpression had features indicative of high malignancy including large size, high histological grade and absence of steroid hormone receptors [8]. However, contrary to the situation in humans, where 85% to 90% of breast carcinomas with HER-2 protein overexpression have a higher number of copies of the oncogene HER-2, a low percentage of feline carcinomas with HER-2 protein overexpression (16.6%) presented a number of HER-2 copies in the limits of what can be considered gene amplification.

Recent immunohistochemical studies have shown highly variable figures of HER-2 protein expression in feline mammary carcinomas ranging from 36% to 90% of the cases [9-11]. In contrast, more homogeneous results have been obtained by diverse immunohistochemical methods both in human and canine mammary cancers. Thus, 15 to 30% of cases of human breast carcinoma present very high levels (overexpression) of HER-2 protein in the membrane of tumour cells [2]. In the dog, 18% to 35% of mammary carcinomas have been shown to overexpress HER-2 protein [13-16]. Differences in the interpretation of immunohistochemical data could be responsible for the highly heterogeneous figures of HER-2 protein expression in feline mammary carcinomas. Thus, immunostaining was often homogeneously distributed throughout the cytoplasm of tumour cells but was easily distinguisable of the stronger membrane staining (Figure 2B). According to the stringent criteria of the HercepTest, only membrane staining should be considered specific. However, methodology-related problems cannot be excluded. All studies so far reproted are retrospective using archival formalin-fixed, paraffin-embedded tissues [9-11], and differences in antigen preservation may exist. In addition, the relatively low number of cases analysed in each series, ranging from 30 [10] to 47 [9], should also be taken into account.

Alterations of the HER-2 oncogene in human breast carcinoma correlate with poor prognosis [3,21]. This has been also the case in the 2 studies that have analyzed this issue in dogs with mammary carcinoma [14,15]. In cats, Millanta and coworkers found correlation between HER-2 protein overexpression in 56% of mammary carcinomas and shorter survival times but not with histological grade of malignancy [9]. The number of studies addressing this issue is too low to draw significant conclusions.

Diverse molecular methods have shown that, in a large majority of human breast carcinomas, HER-2 protein overexpression occurs as a consequence of an alteration in proto-oncogene expression (amplification) that transforms the gene into an oncogene [2,3]. A significant correlation between protein expression and HER-2 mRNA levels has been found in feline [11] as well as in canine [12] mammary carcinomas. However, the number of HER-2 copies was normal in both canine [14] and feline [11] cases using chromogenic *in situ *hybrydization and quantitative PCR, respectively. In this study, 2 mammary carcinomas with HER-2 overexpression had between 5 and 10 copies/nucleus in some 50% of the tumour cells. Although still higher than normal, this level of HER-2 amplification is considered doubtful in human breast cancer and requires to be distinguished from chromosomal aneuploidy [3]. For this, fluorescence or chromogenic *in situ *hybridization of chromosome 17 centromere probes is performed. Proto-oncogene HER-2 maps to chromosomes 17 and 1 in man and dog, respectively, but its chromosome location is not known in the cat. Accordingly, feline mammary carcinomas with HER-2 protein overexpression show normal HER-2 copy number and would be a suitable natural model of the 10–15% of human breast carcinomas with HER-2 protein overexpression without gene amplification.

## Conclusion

As human breast cancer, a subset of feline mammary cancer overexpress HER-2 protein and have signs indicative of worse prognosis, although future multivariate prognostic studies should confirm this finding. Contrary to the human neoplasm, however, HER-2 protein overexpression is not associated with gene amplification. For this reason, feline mammary cancer would be a suitable natural model of that subset of human breast carcinomas with HER-2 protein overexpression without gene amplification.

## Competing interests

The author(s) declare that they have no competing interests.

## Authors' contributions

JO performed all the immunohistochemical and in situ hybridization studies of the oncogene HER-2, performed tumour size measurements and histologic grade of the tumours, participated in the analysis of data and first drafted the manuscript. YM performed the steroid hormones receptors immunohistochemical assays and participated in the study of the histologic grade. RD was responsible for data analysis and performed the statistical study. CR participated in the study design and drafting of the manuscript. JMM conceived of the study, was responsible for its design, participated in data analysis and in the draft of the manuscript and coordinated the whole work. All authors read and approved the final manuscript

## Pre-publication history

The pre-publication history for this paper can be accessed here:


